# Severe Respiratory Illness and Death Associated with Outbreak of Human Rhinovirus B14 among Older Adults, France, 2024

**DOI:** 10.3201/eid3205.250981

**Published:** 2026-05

**Authors:** Julien Andreani, Céline Boschi, Anne Decoppet, Jeremy Delerce, Gwilherm Penant, Aylin Karadeniz, Clio Grimaldier, Priscilla Jardot, Laure Zangoli, Maud Mandy, Nicole Vigroux, Florent Polesso, Sophie Edouard, Philippe Cano, Jean-Christophe Lagier, Bernard La Scola, Philippe Colson

**Affiliations:** Institut hospitalo-universitaire Méditerranée Infection, Marseille, France (J. Andreani, C. Boschi, J. Delerce, G. Penant, A. Karadeniz, C. Grimaldier, P. Jardot, S. Edouard, J.-C. Lagier, B. La Scola, P. Colson); Aix-Marseille University, Marseille (J. Andreani, C. Boschi, J. Delerce, S. Edouard, J.-C. Lagier, B. La Scola, P. Colson); Assistance Publique-Hôpitaux de Marseille, Marseille (J. Andreani, C. Boschi, G. Penant, A. Karadeniz, C. Grimaldier, P. Jardot, J.-C. Lagier, B. La Scola, P. Colson); Agence Régionale de la Santé Provence-Alpes Côte d’Azur, Marseille (A. Decoppet, P. Cano); Centre Hospitalier Intercommunal de Brignoles–Le Luc, Brignoles, France (L. Zangoli, M. Mandy, F. Polesso); Laboratoire de Biologie Médicale Eurofins, Garéoult, France (N. Vigroux)

**Keywords:** Human rhinovirus, rhinovirus B14, respiratory infections, viruses, outbreak, elderly people, next-generation sequencing, metagenomics, France

## Abstract

We investigated an outbreak of unknown respiratory disease and 8 deaths among older adults in a long-term care facility in France. We identified human rhinovirus (HRV) by quantitative PCR and HRV-B14 by metagenomics. We obtained 5 HRV-B14 genomes that diverged from 5 publicly available genomes. Real-time metagenomics could enable rapid clinical diagnoses.

Viral respiratory infections can be particularly severe in fragile persons, especially older adults (*1*). Influenza virus and respiratory syncytial virus (RSV) are known to be associated with high mortality rates among persons >65 years of age (*2*). However, other respiratory viruses, such as human rhinovirus (HRV), also can lead to hospitalization and death (*3*). HRV could become a predominant viral etiology now that effective vaccines are available to prevent severe outcomes from influenza and RSV among older adults (*4*). 

During late fall 2024, a total of 18 of 66 older adult residents of a long-term care facility (LTCF) in southeastern France developed severe respiratory illnesses. Five patients had rapid onset cardiorespiratory decompensation and died (Figure 1), which prompted notification to health authorities at the Agence Régionale de la Santé Provence-Alpes Côte d’Azur (Marseille, France). Results of quantitative PCR (qPCR) for SARS-CoV-2, influenza viruses, and RSV were negative. 

**Figure 1:**
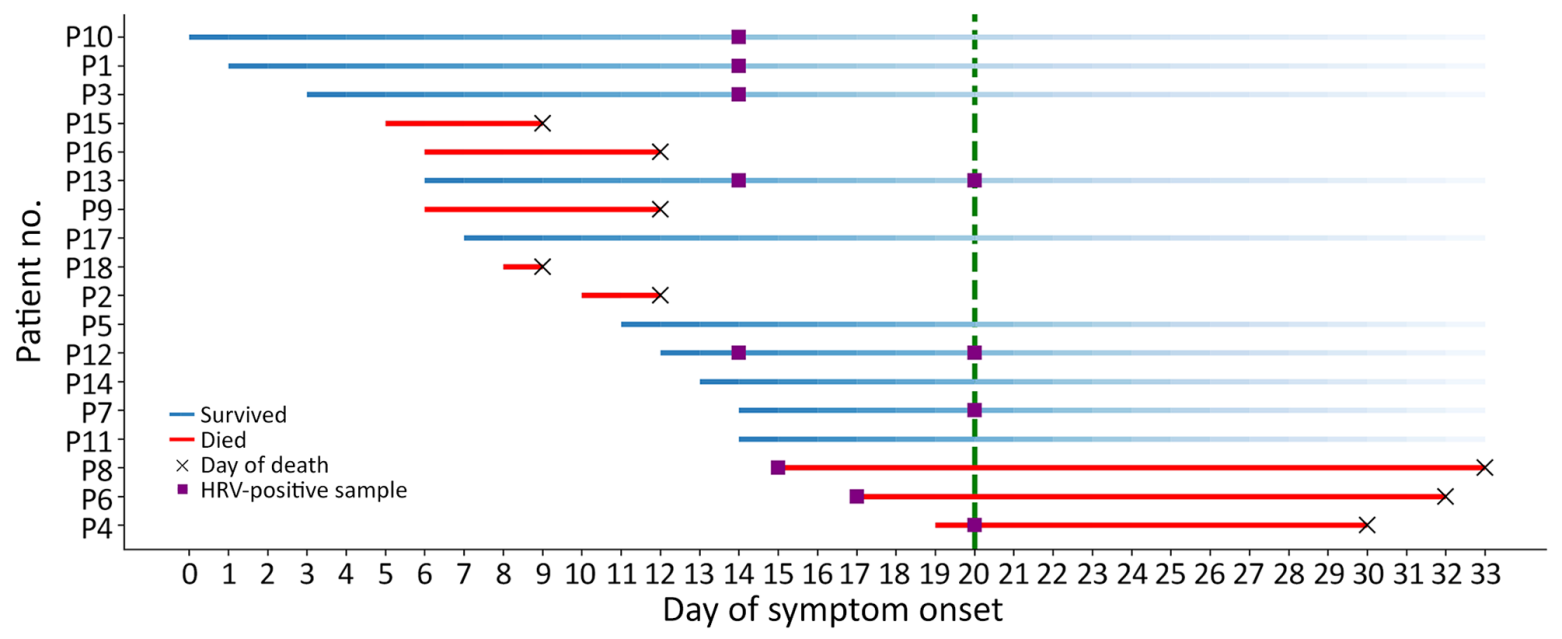
Timeline of severe respiratory illness and death associated with outbreak of HRV-B14 among older adults, France, 2024. Each row represents 1 case and starts on the day of symptom onset, on the basis of retrospectively collected information. Crosses indicate timeline for death. Purple squares indicate HRV RNA–positive samples. The green vertical dotted line indicates transfer of clinical samples to the Marseille laboratory for more extensive diagnosis and metagenomic analyses. Patient 9 had 2 additional HRV-positive postmortem samples (data not shown). No tests were performed for cases P15–P18. No nasopharyngeal swab sample was available for P2. HRV, human rhinovirus.

Health authorities sent residue nasopharyngeal swab samples to the microbiological and virological diagnostic laboratory of the University Hospital Institute (IHU) Méditerranée Infection in Marseille for retrospective testing. We report the rapid multiplex qPCR and metagenomic investigation of that cluster of severe and fatal respiratory infections.

## The Study

At IHU, we initially received 11 nasopharyngeal samples collected 3–10 days earlier from 11 of the LTCF patients. We first attempted virus isolation in a Biosafety Level 3 laboratory, as previously described (*5*). Within 3 hours of receipt, we tested samples by FilmArray Respiratory Panel 2 multiplex qPCR (BioFire Diagnostics, https://www.biofiredx.com); 4 samples tested HRV RNA–positive. All attempted cultures were negative. 

The next day, we extracted DNA and RNA for next-generation sequencing (NGS) (Appendix). We performed qPCR on nucleic acid extracts by using the FTD Respiratory Pathogens 21 Assay (Fast Track Diagnostics/Siemens Healthineers, https://www.siemens-healthineers.com) and in-house designed qPCR (Appendix). Results of qPCR targeting 46 pathogens were negative for all but 1 sample, which was *Staphylococcus aureus* DNA–positive. 

We then performed NGS on DNA and RNA from the 4 HRV-positive, 2 additional HRV-negative samples, and a negative, sample-free control by using the Ligation Sequencing Kit for library preparation and sequencing on GridION (Oxford Nanopore Technologies, https://nanoporetech.com). Next, we sequenced on the MiSeq platform using MiSeq Reagent version 2 kit (Illumina Inc., https://www.illumina.com), according to manufacturer’s protocol (Appendix). 

We conducted bioinformatic analyses of generated NGS reads by using various commercial and freely available tools, as well as in-house pipelines (Appendix). Six hours after starting NGS, 15 NGS reads generated from the samples of 2 patients (13 reads from 1 patient and 2 reads from the other), ranging from 468 to 2,024 nt in length, were found to be best matches with an HRV-B14 genome (GenBank accession no. NC_001490.1) (Table; Appendix Tables 1, 2). We used those reads to obtain consensus sequences, one of 4,447 nt in 3 contigs for one sample, and the other of 1,019 nt for the other sample (Appendix). The negative control had no HRV reads.

**Table:** Clinical outcomes and results of molecular testing of nasopharyngeal swab samples collected in a study of severe respiratory illness and death associated with outbreak of HRV-B14 among older adults, France, 2024*

Pt. no.	Sample no.	Biofire qPCR†	FTD multiplex qPCR (Ct)‡	Shotgun mNGS		Probe-based mNGS; % coverage§	VP1 genotype	Hospitalized; clinical outcome
				First batch	Second batch			
1	1	HRV	–	HRV-B14; 2 reads	ND	HRV-B14; 73	HRV-B14	N; recovered
2	NS	ND	ND	ND	ND	ND	ND	Y; died
3	3	HRV	HRV (31)	HRV-B14; 13 reads	ND	HRV-B14; 81	HRV-B14	N; recovered
4	7	HRV	–	ND	No HRV detected	ND	ND	N; died
5	8	–	–	ND	No HRV detected	ND	–	N; recovered
6#	9	HRV	–	ND	No HRV detected	HRV-B14; 13	–	Y; died
7	14	HRV	ND	ND	No HRV detected	ND	ND	N; recovered
8	15	HRV	HRV (31)	No HRV detected	ND	HRV-B14; 43	HRV-B14	Y; died
9**	18	HRV	–	No HRV detected	ND	ND	–	Y; recovered
	22	–	–	ND	No HRV detected	ND	ND	
	23	HRV	–	ND	ND	ND	ND	
10	24	HRV	–	ND	No HRV detected	ND	–	N; recovered
	28	–	–	ND	No HRV detected	ND	ND	
11††	29	–	–	No HRV detected	ND	ND	–	N; recovered
12	30	HRV	HRV (33)	No HRV detected	ND	HRV-B14; 32	HRV-B14	N; recovered
	34	HRV	HRV (34)	ND	No HRV detected	ND	ND	
13	35	HRV	–	ND	No HRV detected	ND	–	N; recovered
	39	HRV	–	ND	No HRV detected	ND	ND	
	40	–	–	ND	ND	ND	ND	
14	42	–	–	ND	No HRV detected	ND	–	Y; recovered

*Ct, cycle threshold; HRV, human rhinovirus; mNGS, metagenomic next-generation sequencing; ND, not done; NS, no sample; Pt., patient; qPCR, quantitative PCR; VP1, viral capsid-encoding gene; –, negative. †FilmArray Respiratory Panel 2 multiplex qPCR assay (BioFire Diagnostics, https://www.biofiredx.com). ‡FTD Respiratory Pathogens 21 (Fast Track Diagnostics/Siemens Healthineers, https://www.siemens-healthineers.com). §Probe-based ± ARTIC-like viral enrichment, then mNGS. #Patient had a history of oxygen requirement and metastatic bladder cancer. **Patient was anorexic and on an intravenous drip. ††Patient had recent anorexia and cough develop.

We designed in-house PCR systems by using the Primer3Plus web application (https://www.primer3plus.com/index.html) to fill gaps in reconstructed HRV-B14 genomes (Appendix Table 3). Then, we used IQ-TREE (http://www.iqtree.org) to select the best model for each alignment and built maximum-likelihood phylogenetic trees. We visualized trees incorporating all complete HRV-B14 genomes by using iTOL version 7 (https://itol.embl.de).

Five days after we received the initial samples, we received additional samples from the LTCF outbreak; in total, multiplex qPCRs eventually detected HRV RNA in 13/19 nasopharyngeal swab samples from 10/13 patients. Eight samples tested positive with the BioFire assay and negative with the FTD assay (Table; Appendix Table 2). An HRV-B14–specific qPCR we designed from partial genomes was positive for 5 patients (Appendix Table 3).

**Figure 2:**
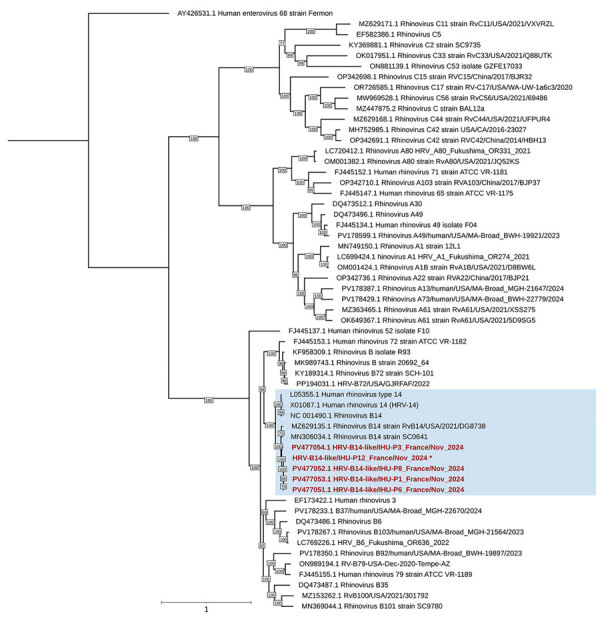
Phylogenetic tree of HRV-B14 from outbreak of severe respiratory illness and death among older adults, France, 2024. Tree includes HRV genomes downloaded from GenBank; accession numbers are indicated. Red font indicates genomes obtained in this study; blue background indicates HRV-B14 genomes. Asterisk (*) indicates that the genome is fragmented (multiple GenBank accession numbers available). Genome alignment was performed by using MUSCLE (https://www.ebi.ac.uk/Tools/msa/muscle) with standard parameters for nucleotide sequences. Tree was built by using IQ-TREE (http://www.iqtree.org) with the transitional model 2 plus empirical base frequencies plus proportion of invariable site plus gamma-distributed rate variation with 4 categories model identified as the most suitable by the best-fit model research module according to the Bayesian information criterion. A total of 10,000 ultra-fast bootstrap replicates were performed. Scale bar indicates nucleotide substitutions per site. HRV, human rhinovirus.

Pre-NGS viral nucleic acid enrichment using the Respiratory Viral Research Panel (Twist Bioscience, https://www.twistbioscience.com) enabled us to obtain 5 partial HRV-B14 genomes from 5 patients. Genomes were 913–6,644 nt in length and 13%–86% of the GenBank reference genome (accession no. NC_001490.1). In the phylogenetic tree, those 5 genomes clustered together with 100% bootstrap value near 5 other genomes available in public databases (Figure 2). However, 1 genome from patient P3 stood apart; its nucleotide similarity was 97.1%–98.1% with the 4 other genomes, whereas those 4 genomes had 99.9%–100.0% similarity to each other. Sequencing of the HRV virus capsid protein (VP) 1–encoding gene confirmed HRV-B14 for 4 of the initial 11 samples tested from the LTCF (Table). 

**Figure 3:**
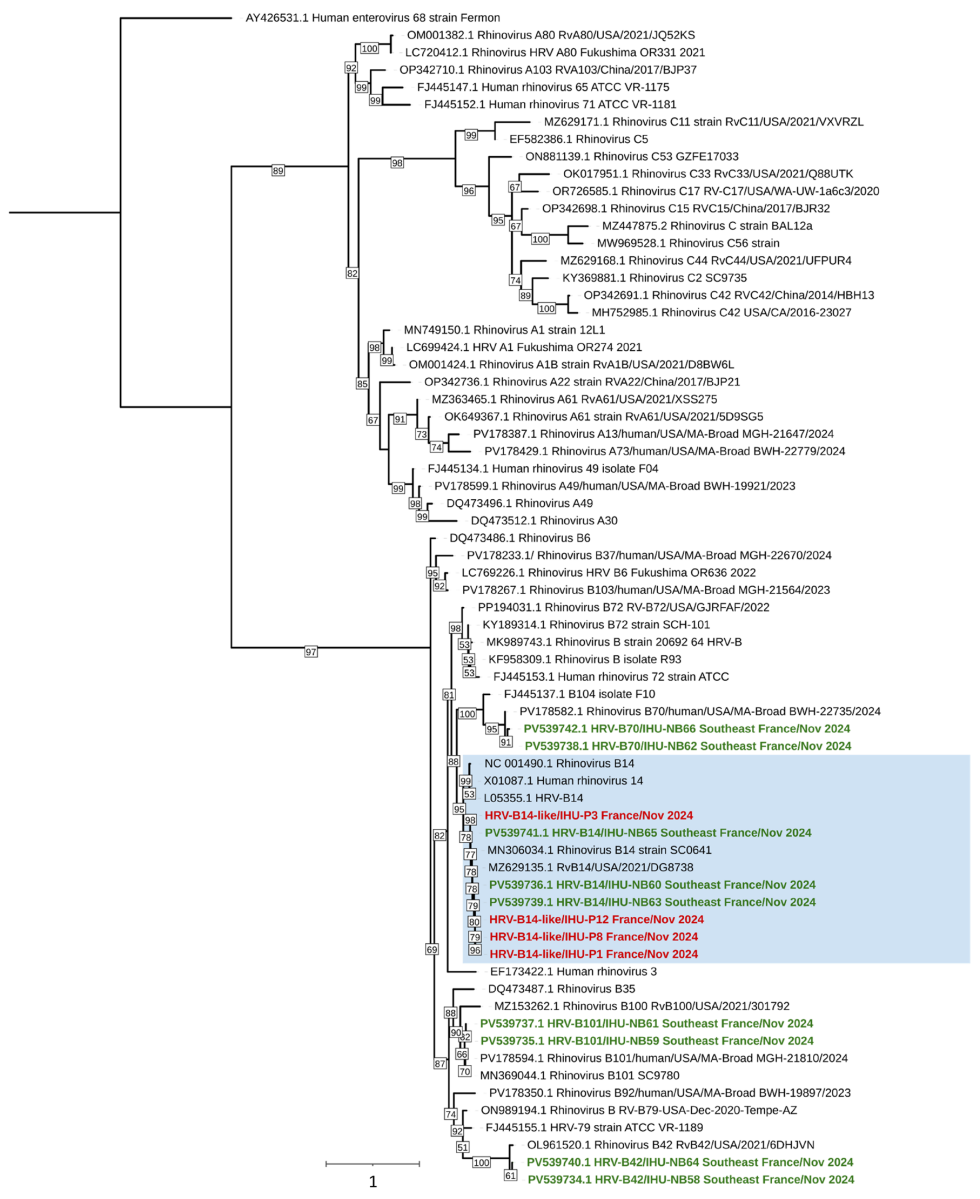
Phylogenetic tree of HRV-B sequences circulating in Marseille during severe respiratory illness and death associated with outbreak of HRV-B14 among older adults, France, 2024. Viral protein 1–encoding gene sequence alignment was performed by using MUSCLE (https://www.ebi.ac.uk/Tools/msa/muscle) with standard parameters for nucleotide sequences. Blue background indicates the HRV-B14 sequences; red font indicates sequences obtained from the cluster of cases among older adults; green font indicates sequences obtained from other HRV RNA–positive samples detected in our laboratory during the same timeframe as the outbreak. All others sequences were downloaded from GenBank; accession numbers are indicated. The sequence obtained from patient P6 does not appear in this tree because it was a partial sequence and does not align with the genome region used in this analysis. The IQ-TREE software was used to build the tree with the general time-reversible model plus empirical base frequencies plus FreeRate model with 10 rate categories model identified as the most suitable by the best-fit model research module according to the Bayesian information criterion. A total of 10,000 ultra-fast bootstrap replicates were performed. Scale bar indicates nucleotide substitutions per site. HRV, human rhinovirus.

We also tested 86 residue nasopharyngeal swab samples collected in southeastern France as part of routine diagnostic testing during the same timeframe as the cases from the LTC facility during late autumn 2024. Viral capsid VP1–encoding virus genotyping for all 86 nasopharyngeal swab samples identified 9 additional HRV-B sequences, 3 of which were HRV-B14 on the basis of BLAST (https://blast.ncbi.nlm.nih.gov) similarity and phylogeny (Figure 3). 

The outbreak among LTCF residents resulted in 18 severe illnesses, from which 8 (44%) patients died. Of note, metagenomic methods enabled us to identify HRV-B14 within ≈30 hours of receiving samples; without those methods, the cause of the outbreak would have largely remained elusive. 

Viral nucleic acid enrichment successfully lengthened the HRV-B14 genomes from the LTCF outbreak, 1 of which greatly differed from the 4 others, indicating >2 closely related HRV-B14 strains were circulating during this outbreak. In addition, HRV-B14 was detected in 3% of HRV-positive samples received at our laboratory during the same period as the LTCF outbreak, indicating that HRV-B14 circulated in that geographic area in fall of 2024. 

Previous metagenomics-based approaches have successfully identified various pathogens during outbreak investigations (*6*–*10*). For instance, in the United States, metagenomics used on 6 nasopharyngeal swab samples from 4 case-patients in a pulmonary ward and on 10 nasopharyngeal swab samples from 9 control outpatients enabled investigators to determine that case-patients’ HRV sequences were not genetically related, dispelling concerns of single-source nosocomial transmission <24 hours of receiving samples (*7*). That case demonstrates the benefits of metagenomics in routine settings.

During the LTCF outbreak, *S. aureus* DNA was the only other pathogen detected by qPCR, and only in 1 sample. Notwithstanding that finding, co-infections might have been missed because specific qPCR, serologic, or culture assays were not used for all possible infectious agents; because viral or microbial loads were too low for detection; or because our laboratory was not involved in first-line diagnosis and the time between respiratory sample collection and testing at our laboratory was up to 10 days. 

## Conclusions

Severe and life-threatening HRV infections previously have been reported among older persons (*11*,*12*). Illness severity as assessed through rates of hospital admission, intensive care unit admission, and death in those studies were similar to those previously reported among immunocompromised adults infected with the 2009 influenza A(H1N1) pandemic virus (*11*,*12*) or to rates among hematopoietic cell transplant recipients with RSV or influenza virus infection (*13*,*14*). We speculate that HRV could lead to severe disease and death in fragile older persons, as seen with RSV (*1*), but data are scarce. Moreover, those prior assessments did not address whether severity differed between viral genotypes, and no specific data on HRV-B14 epidemiology, infectiousness, or clinical severity are available. 

In summary, our findings indicate >1 HRV-B14 strain was circulating and caused severe illness and death among a population of older adults in southeastern France. Rapid identification (≈30 hours) of the viral etiology in this case demonstrates that expanded use of real-time metagenomics for routine diagnostic testing in clinical virology laboratories, particularly targeting respiratory viruses other than vaccine-preventable influenza viruses and RSV, could enable early detection and management of such respiratory pathogens.

Appendix Additional information on severe respiratory illness and death associated with outbreak of human rhinovirus B14 among older adults, France, 2024.
