# Supplementary material for: Severe Respiratory Illness and Death Associated with Outbreak of Human Rhinovirus B14 among Older Adults, France, 2024
Source: Emerg Infect Dis. 2026 May;32(5):768–73. doi: 10.3201/eid3205.250981 (PMC13174967; doi:10.3201/eid3205.250981)
Supplement: Appendix — Additional information on severe respiratory illness and death associated with outbreak of human rhinovirus B14 among older adults, France, 2024. [file 25-0981-Techapp-s1.pdf]

Article DOI: https://doi.org/10.3201/eid3205.250981

EID cannot ensure accessibility for supplementary materials supplied by authors. Readers who have difficulty accessing supplementary content should contact the authors for assistance.

Severe Respiratory Illness and Death Associated with Outbreak of Human Rhinovirus B14 among Older Adults, France, 2024

Appendix

Supplementary Methods

Patients and clinical samples

Clinical samples were sent by the Regional Health Agency of the Provence-Alpes-Côte d'Azur region to the microbiological and virological diagnostic laboratory and the infectious diseases crisis unit of university hospital institute (IHU) Méditerranée Infection and public and university hospitals of Marseille (AP-HM) in southeastern France. This aimed at investigating and identifying the causative agent(s) in a cluster of cases of respiratory diseases that occurred during late autumn 2024 among residents of a residential facility for dependent elderly people in the south-east of France. Residues of nasopharyngeal samples that had been collected from 13 of these patients were received in two batches. A first series consisted of 11 samples from 11 patients that had been previously collected for diagnostic purposes and sent to a private medical biology laboratory, and that were received at our institute between 3 and 10 days post-sampling. The second series was received 5 days later and consisted of two samples from two additional patients plus six samples from four patients for whom samples were already received in the first batch. Additional data regarding clinical samples received at IHU Méditerranée Infection are provided in Appendix Tables 1 and 2.

Culture inoculation for virus or microorganism isolation

Viral culture isolation was attempted at the biosafety level 3 (BSL-3) laboratory of our institute using residues from these nasopharyngeal swabs, although these were inappropriate for

viral culture, and on blood and urine, on a cell panel at 37°C under 5% CO_2 atmosphere, as previously described (*1*).

Real-time PCR

All clinical samples were initially handled at the BSL-3 laboratory of our institute. The first microbiological and virological analyses were carried out retrospectively on residues of nasopharyngeal swabs within three hours of their receipt. qPCR were performed using the FilmArray multiplex Respiratory panel 2 (Biofire, bioMérieux, Marcy-l'Etoile, France). Also, DNA and RNA were extracted from 200 µL of these respiratory samples using the MagMAX viral/pathogen II kit on a Kingfisher flew purification system 96 instrument (Thermo Fisher Scientific, Waltham, MA, USA) or the EZ2 Virus Mini Kit v2.0 on a BioRobot EZ2 workstation (Qiagen, Hilden, Germany). Then nucleic acid extracts were tested by qPCR using the Fast Track Diagnostics Respiratory Pathogens 21 (Fast Track Diagnostics, Luxembourg), as well as with in-house designed qPCR targeting *Chlamydia psittaci*, *Chlamydia pneumoniae*, *Mycobacterium tuberculosis*, *Coxiella burnetii*, *Streptococcus pyogenes*, *Streptococcus pneumoniae*, *Haemophilus influenzae, Legionella pneumophila*, and *Staphylococcus aureus*, as previously described (*2*).

Metagenomic next-generation sequencing and bioinformatic analyses

Primarily, next-generation sequencing (NGS) was performed directly from the DNA/RNA extract and from cDNA generated by reverse transcription using the SuperScript VILO cDNA synthesis kit (Thermo Fisher Scientific) then second strand synthesis with the Klenow large fragment DNA polymerase I (New England Biolabs, Beverly, MA, USA). NGS was carried out using the Oxford Nanopore technology (ONT) with the Ligation Sequencing Kit (SQK-LSK109) for library preparation then loading on a flow cell Mk I, R10 (GridION instrument, Oxford Nanopore Technologies, Oxford, UK). Secondarily, NGS was performed using the Illumina technology with the Nextera XT protocol and the MiSeq Reagent v2 kit (Illumina, San Diego, USA), after an enrichment procedure in viral nucleic acid using the Twist Respiratory Viral Research Panel (Twist Bioscience, San Francisco, USA), according to the manufacturer's protocol. A total of 4 nM of the amplified library was loaded on an Illumina MiSeq instrument for a 39-hour run with 20% PhiX and a reading of 2x250 nt with the paired-end strategy (Illumina).

Primarily, the analysis of shotgun metagenomic data obtained by NGS was performed (i) by a mapping of NGS reads with the CLC Genomics software (https://digitalinsights.qiagen.com/) against an in-house database of genomes of respiratory viruses collected from GenBank (*3*) (https://www.ncbi.nlm.nih.gov/genbank/) and from the BC-ViPR database (*4*) (https://www.bv-brc.org/); and (ii) using the Kraken2 version 2.1.3 software (*5*) with a confidence threshold of 0.01 against the standard Kraken 2/Bracken Refseq database dating back to 04/09/2024 (https://benlangmead.github.io/aws-indexes/k2). Secondarily, base-calling for ONT NGS data was performed using Medaka 1.11.3 (https://github.com/nanoporetech/medaka/releases) and Longshot 0.4.1 (*6*). Consensus sequences were generated with BCFtools v.1.18 (*7*). Mapping of NGS reads against human rhinovirus (HRV) B14 reference genome (NC_001490.1) was performed using Minimap2 v.2.28-r1209 (*8*). The SAM file was filtered with samtools v.1.18 and processed with align_trim to remove incorrect amplicon pairs. In case a consensus was obtained for a same clinical sample using both NGS technologies, the consensus sequence was mapped against the same reference, and a final consensus was generated with Samtools consensus.

Thereafter, PCR systems were in-house designed using the Primer3Plus web application (*9*) (https://www.primer3plus.com/index.html) to fill the gaps remaining in reconstructed HRV-B14 genomes with primers targeting the regions flanking these gaps (Appendix Table 3). Five µL of RNA extract were used with these PCR systems and the SuperScript III One Step RT-PCR kit (Invitrogen, Carlsbad, CA, USA), to produce PCR amplicons. The same HRV-B14 genome reconstruction strategy than described above was then used, incorporating previously obtained contigs as well as PCR amplicons recovered by NGS with the ONT.

HRV-B14-specific qPCR system

A HRV-B14-specific qPCR system was designed based on sequences from the two first partial viral genomes obtained, using PrimerPplus web application (https://www.primer3plus.com/index.html) (Appendix Table 3). RNA/DNA extracts priorly obtained from nasopharyngeal samples were tested with the Fast One-step kit and this qPCR system and final concentrations of primers and probe of 500 nM and 250 nM, respectively, on a QS5 thermocycler (Thermo Fisher Scientific).

Phylogenetic analysis

Using the consensus genome sequence reconstructed with the different NGS approaches, a BLASTn search (https://blast.ncbi.nlm.nih.gov/Blast.cgi) was performed to recover all complete HRV-B14 genomes. Genomes of HRV-A and B genotypes and a complete enterovirus genome serving as outgroup sequence were incorporated in the phylogeny reconstruction. Sequence alignment was performed using the Muscle program with standard parameters (*10*), then alignments were visualized and manually curated with Jalview (*11*). Finally, IQ-TREE was used to select the best model for each alignment and to build maximum-likelihood phylogenetic trees (*12*), which were visualized using Itol v7 (*13*).

Genotyping of HRV from HRV RNA-positive nasopharyngeal samples collected in our institution

A total of 86 residues of nasopharyngeal samples collected during late autumn 2024 that had been sent to our laboratory for the purpose of diagnosis of agents of respiratory infections, and had been found HRV RNA-positive using the FilmArray multiplex Respiratory panel 2 or the Fast Track Diagnostics Respiratory Pathogens 21, were tested retrospectively to amplify a fragment of the virus capsid gene for HRV genotyping (Table; Appendix Tables 1 and 2). DNA/RNA was extracted from 200 µL of these samples with the MagMAX viral/pathogen II kit on a Kingfisher instrument (Thermo Fisher Scientific). Then, first round and nested PCR were performed as previously described (*14*). First round was carried out from 5 µl of nucleic acid extract using the Superscript III/Platinum Taq one-step rRT-PCR kit (Invitrogen) with primers at a final concentration of 0.2 µM in a final volume of 25 µL. Thereafter, nested-PCR was carried out using 5 µL of first round PCR product. NGS of obtained PCR amplicons was performed using the ONT.

Supplementary Results

Residues of samples from 14 of these patients were sent by the Regional Health Agency to our laboratory for retrospective testing to investigate these cases, including nasopharyngeal swabs from 13 patients. During the afternoon of the day following the request of the Regional Health Agency, the IHU laboratory received residues from 11 nasopharyngeal samples, each from a different patient, collected between 3 and 10 days before their receipt at our laboratory. Within three hours of their receipt, all nasopharyngeal samples were tested by multiplex qPCR

using the FilmArray Respiratory panel 2 (Table 1; Appendix Tables 1 and 2). This allowed detecting four samples from four patients as HRV RNA-positive. All attempted cultures were negative. The day after, DNA/RNA metagenomics was performed for six samples including these four HRV RNA-positive samples by the ONT, directly from the DNA/RNA extracts. Manipulations were started at 8 a.m. and after six hours of the NGS run, reads generated were analyzed by mapping to an in-house genome sequence database previously created with multiple respiratory viruses, and concurrently using the Kraken2 tool. These two analyses made it possible to highlight for two of the six samples the presence of a very small number of reads, 13 and 2, best matching with a HRV-B14 genome. These reads had a size ranging from 468 to 2,024 nt, and allowed obtaining consensus sequences with a total size of 4,447 nt in three contigs and of 1,019 nt, respectively. No HRV reads were present in the negative control sample. A similarity search conducted using the BLASTn web application only retrieved five HRV-B14 genomes in the GenBank database. At this stage, ≈30 hours post-receipt of the samples at our laboratory, it was possible to suspect a HRV-B14 outbreak in the residential facility for dependent elderly people. Overall, qPCR carried out using both the BioFire and the FTD assays enabled detecting HRV RNA in 13 of 19 nasopharyngeal swab residues from 10 of 13 different patients for whom samples were tested. Eight samples tested positive with the Biofire assay, which uses nested PCR that is deemed to enhance detection sensitivity, and negative with the FTD assay (Table). A HRV-B14-specific qPCR in-house designed based on obtained partial genomes was used to test respiratory samples from the 13 patients and was positive for five of them (samples 1, 3, 15, 30 and 34) including four from whom viral genomes had been obtained.

In parallel, virus nucleic acid enrichment pre-NGS allowed obtaining five partial genomes of HRV-B14 from the nasopharyngeal samples from five different patients. These partial genomes were between 913 and 6,644 nt in size, which corresponds to a coverage of 13 to 86% of HRV genome GenBank accession no. NC_001490.1. They were clustered together (bootstrap value, 100%), apart from the five other genomes available in public databases (Figure 2). However, one of them (from patient P3) stood apart from the four other genomes, being more genetically divergent. Indeed, nucleotide similarity between this genome and the four others was 97.1% with P1, 97.3% with P6, 97.2% with P8 and 98.1% with P12, whereas nucleotide similarity ranged between 99.9% and 100.0% between P1, P6, P8 and P12 genomes. Later, conventional PCR targeting the VP1 gene encoding the capsid of HRV genotypes B followed by

sequencing confirmed the presence of HRV-B14 for 4 of the 11 samples tested, including for one sample (from P3) found negative by qPCR with the Biofire assay (Table, Figure 3). Finally, we evaluated by sequencing-based genotyping whether HRV-B14 was also present in nasopharyngeal samples tested at our laboratory as part of routine diagnosis during the same period of time than for cases. Nine (10%) of 86 HRV-positive samples were positive with an HRV-B. Sequencing with the ONT of the PCR amplicons then BLAST similarity search and phylogeny revealed that three of these HRV-B were classified as B14, while two each were classified as B42, B70, or B101 (Figure 3). Besides, blood samples were tested with the multiplex qPCR FilmArray Biothreat panel assay (bioMérieux, Marcy-l'Etoile, France) without any positive results (Table; Appendix Tables 1 and 2). Finally, attempts of culture isolation of viruses and microorganisms were negative from all samples, considering nasopharyngeal swabs were inappropriate for viral culture.

References

1. Wurtz N, Penant G, Jardot P, Duclos N, La Scola B. Culture of SARS-CoV-2 in a panel of laboratory cell lines, permissivity, and differences in growth profile. Eur J Clin Microbiol Infect Dis. 2021;40:477–84. PubMed https://doi.org/10.1007/s10096-020-04106-0

2. Morel AS, Dubourg G, Prudent E, Edouard S, Gouriet F, Casalta JP, et al. Complementarity between targeted real-time specific PCR and conventional broad-range 16S rDNA PCR in the syndrome-driven diagnosis of infectious diseases. Eur J Clin Microbiol Infect Dis. 2015;34:561–70. PubMed https://doi.org/10.1007/s10096-014-2263-z

3. Sayers EW, Cavanaugh M, Clark K, Pruitt KD, Sherry ST, Yankie L, et al. GenBank 2024 update. Nucleic Acids Res. 2024;52(D1):D134–7. PubMed https://doi.org/10.1093/nar/gkad903

4. Olson RD, Assaf R, Brettin T, Conrad N, Cucinell C, Davis JJ, et al. Introducing the Bacterial and Viral Bioinformatics Resource Center (BV-BRC): a resource combining PATRIC, IRD and ViPR. Nucleic Acids Res. 2023;51(D1):D678–89. PubMed https://doi.org/10.1093/nar/gkac1003

5. Wood DE, Lu J, Langmead B. Improved metagenomic analysis with Kraken 2. Genome Biol. 2019;20:257. PubMed https://doi.org/10.1186/s13059-019-1891-0

6. Edge P, Bansal V. Longshot enables accurate variant calling in diploid genomes from single-molecule long read sequencing. Nat Commun. 2019;10:4660. PubMed https://doi.org/10.1038/s41467-019-12493-y

7. Danecek P, Bonfield JK, Liddle J, Marshall J, Ohan V, Pollard MO, et al. Twelve years of SAMtools and BCFtools. Gigascience. 2021;10:giab008. PubMed https://doi.org/10.1093/gigascience/giab008

8. Li H. Minimap2: pairwise alignment for nucleotide sequences. Bioinformatics. 2018;34:3094–100. PubMed https://doi.org/10.1093/bioinformatics/bty191

9. Untergasser A, Nijveen H, Rao X, Bisseling T, Geurts R, Leunissen JA. Primer3Plus, an enhanced web interface to Primer3. Nucleic Acids Res. 2007;35:W71–4. PubMed https://doi.org/10.1093/nar/gkm306

10. Edgar RC. MUSCLE: multiple sequence alignment with high accuracy and high throughput. Nucleic Acids Res. 2004;32:1792–7. PubMed https://doi.org/10.1093/nar/gkh340

11. Waterhouse AM, Procter JB, Martin DMA, Clamp M, Barton GJ. Jalview version 2—a multiple sequence alignment editor and analysis workbench. Bioinformatics. 2009;25:1189–91. PubMed https://doi.org/10.1093/bioinformatics/btp033

12. Minh BQ, Schmidt HA, Chernomor O, Schrempf D, Woodhams MD, von Haeseler A, et al. IQ-TREE 2: new models and efficient methods for phylogenetic inference in the genomic era. Mol Biol Evol. 2020;37:1530–4. PubMed https://doi.org/10.1093/molbev/msaa015

13. Letunic I, Bork P. Interactive Tree of Life (iTOL) v6: recent updates to the phylogenetic tree display and annotation tool. Nucleic Acids Res. 2024;52(W1):W78–82. PubMed https://doi.org/10.1093/nar/gkae268

14. Mubareka S, Louie L, Wong H, Granados A, Chong S, Luinstra K, et al. Co-circulation of multiple genotypes of human rhinovirus during a large outbreak of respiratory illness in a veterans' long-term care home. J Clin Virol. 2013;58:455–60. PubMed https://doi.org/10.1016/j.jcv.2013.06.037

Appendix Table 1. Main results obtained from microbiological tests (real-time PCR, metagenomics and culture)

Patient no.	Sample no.	Nature	Biofire multiplex qPCR respiratory panel	Biofire Biothreat qPCR panel	FTD multiplex qPCR respiratory panel (Ct)	Bacterial qPCR (Ct)	Shotgun mNGS (first batch)	Shotgun mNGS (second batch)	Probe-based±ARTIC-like viral enrichment then mNGS	GenBank accession number	HRV VP1 genotyping	Virus culture
1	1	Nasopharyngeal swab	HRV		Neg.	Neg.	HRV-B14 (2 reads)		HRV-B14 (73% of the whole genome)	PV477053	HRV-B14	Neg.
2	2	Whole blood (in EDTA-containing tube)		Neg.								Neg.
3	3	Nasopharyngeal swab	HRV		HRV (31)	Neg.	HRV-B14 (13 reads)		HRV-B14 (81% of the whole genome)	PV477054	HRV-B14	Neg.
4	4	Serum										
	5	Whole blood (in EDTA-containing tube)										
	6	Heparin blood tube										
	7	Nasopharyngeal swab	HRV		Neg.			No HRV detection				Neg.
5	8	Nasopharyngeal swab	Neg.		Neg.	Neg.		No HRV detection			Neg.	Neg.
6	9	Nasopharyngeal swab	HRV		Neg.	Neg.		No HRV detection	HRV-B14 (13% of the whole genome)	PV477051	Neg.	Neg.
	10	Whole blood (in EDTA-containing tube)		Neg.								Neg.
7	11	Serum										
	12	Whole blood (in EDTA-containing tube)										
	13	Whole blood (in heparin-containing tube)										
	14	Nasopharyngeal swab	HRV					No HRV detection				Neg.
8	15	Nasopharyngeal swab	HRV		HRV (31)	S. a. (30)	No HRV detection		HRV-B14 (45% of the whole genome)	PV477052	HRV-B14	Neg.
	16	Whole blood (in EDTA-containing tube)		Neg.								Neg.

Patient no.	Sample no.	Nature	Biofire multiplex qPCR respiratory panel	Biofire Biothreat qPCR panel	FTD multiplex qPCR respiratory panel (Ct)	Bacterial qPCR (Ct)	Shotgun mNGS (first batch)	Shotgun mNGS (second batch)	Probe-based±ARTIC-like viral enrichment then mNGS	GenBank accession number	HRV VP1 genotyping	Virus culture
	17	Urine										Neg.
9	18	Nasopharyngeal swab	HRV		Neg.	Neg.	No HRV detection				Neg.	Neg.
	19	Serum										
	20	Whole blood (in EDTA-containing tube)										
	21	Whole blood (in heparin-containing tube)										
	22	Nasopharyngeal swab	Neg.		Neg.			No HRV detection				Neg.
	23	Nasopharyngeal swab	HRV		Neg.							Neg.
10	24	Nasopharyngeal swab	HRV		Neg.	Neg.		No HRV detection			Neg.	Neg.
	25	Serum										
	26	Whole blood (in EDTA-containing tube)										Neg.
	27	Whole blood (in heparin-containing tube)										
	28	Nasopharyngeal swab	Neg.		Neg.			No HRV detection				Neg.
11	29	Nasopharyngeal swab	Neg.		Neg.	Neg.	No HRV detection				Neg.	Neg.
12	30	Nasopharyngeal swab	HRV		HRV (33)	Neg.	No HRV detection		HRV-B14 (32% of the whole genome)	PV477055, PV477056, PV477057, PV477058	Rhinovirus B14	Neg.
	31	Serum										
	32	Whole blood (in EDTA-containing tube)										Neg.
	33	Serum										
	34	Nasopharyngeal swab	HRV		HRV (34)			No HRV detection				Neg.
13	35	Nasopharyngeal swab	HRV		Neg.	Neg.		No HRV detection			Neg.	Neg.
	36	Serum										
	37	Whole blood (in EDTA-containing tube)										Neg.
	38	Serum										

Patient no.	Sample no.	Nature	Biofire multiplex qPCR respiratory panel	Biofire Biothreat qPCR panel	FTD multiplex qPCR respiratory panel (Ct)	Bacterial qPCR (Ct)	Shotgun mNGS (first batch)	Shotgun mNGS (second batch)	Probe-based±ARTIC-like viral enrichment then mNGS	GenBank accession number	HRV VP1 genotyping	Virus culture
	39	Nasopharyngeal swab	HRV		Neg.			No HRV detection				Neg.
	40	Nasopharyngeal swab	Neg.		Neg.							Neg.
14	41	Whole blood (in EDTA-containing tube)		Neg.								
	42	Nasopharyngeal swab	Neg.		Neg.	Neg.		No HRV detection			Neg.	Neg.
	43	Urine										Neg.

*Cb, Coxiella burnetii; FTD, Fast Track Diagnosis multiplex qPCR respiratory panel; HRV, Human Rhinovirus; mNGS, metagenomics by next-generation sequencing; S. a., Staphylococcus aureus; Neg., negative; VP1, viral capsid-encoding gene. Cycle threshold values (Ct) for real-time PCR are indicated into parentheses

Appendix Table 2. Main results obtained from molecular biology tests on nasopharyngeal samples (qPCR and metagenomics) and clinical outcome*

Patient no.	Sample no.	Biofire multiplex qPCR respiratory panel	FTD multiplex qPCR respiratory panel (Ct)	Bacterial qPCR (Ct)	Shotgun mNGS (first batch)	Shotgun mNGS (second batch)	Probe-based±ARTIC-like viral enrichment then mNGS	Genbank accession number	VP1 genotyping	Clinical outcome
1	1	HRV	Neg.	Neg.	HRV-B14 (2 reads)	N.d.	HRV-B14 (Cov. = 73%)	PV477053	HRV-B14	No hospitalization
2	N.a.	N.d	N.d	N.d	N.d	N.d	N.d	-	N.d	Hospital admission, Death
3	3	HRV	HRV (31)	Neg.	HRV-B14 (13 reads)	N.d.	HRV-B14 (Cov. = 81%)	PV477054	HRV-B14	No hospitalization
4	7	HRV	Neg.	N.d.	N.d.	No HRV detection	N.d.	-	N.d.	No hospitalization, Death
5	8	Neg.	Neg.	Neg.	N.d.	No HRV detection	N.d.	-	Neg.	No hospitalization
6†	9	HRV	Neg.	Neg.	N.d.	No HRV detection	HRV-B14 (Cov. = 13%)	PV477051	Neg.	Hospital admission, Death
7	14	HRV	N.d	N.d.	N.d.	No HRV detection	N.d.	-	N.d.	No hospitalization
8	15	HRV	HRV (31)	*S. aureus* (30)	No HRV detection	N.d.	HRV-B14 (Cov. = 43%)	PV477052	HRV-B14	Hospital admission, Death
9‡	18	HRV	Neg.	Neg.	No HRV detection	N.d.	N.d.	-	Neg.	Hospital admission
	22	Neg.	Neg.	N.d.	N.d.	No HRV detection	N.d.	-	N.d.	-
	23	HRV	Neg.	N.d.	N.d.	N.d.	N.d.	-	N.d.	-
10	24	HRV	Neg.	Neg.	N.d.	No HRV detection	N.d.	-	Neg.	No hospitalization
	28	Neg.	Neg.	N.d.	N.d.	No HRV detection	N.d.	-	N.d.	-

Patient no.	Sample no.	Biofire multiplex qPCR respiratory panel	FTD multiplex qPCR respiratory panel (Ct)	Bacterial qPCR (Ct)	Shotgun mNGS (first batch)	Shotgun mNGS (second batch)	Probe-based±ARTIC-like viral enrichment then mNGS	Genbank accession number	VP1 genotyping	Clinical outcome
11§	29	Neg.	Neg.	Neg.	No HRV detection	N.d.	N.d.	-	Neg.	No hospitalization
12	30	HRV	HRV (33)	Neg.	No HRV detection	N.d.	HRV-B14 (Cov. = 32%)	PV477055-PV477058	HRV-B14	No hospitalization
	34	HRV	HRV (34)	N.d.	N.d.	No HRV detection	N.d.	-	N.d.	-
13	35	HRV	Neg.	Neg.	N.d.	No HRV detection	N.d.	-	Neg.	No hospitalization
	39	HRV	Neg.	N.d.	N.d.	No HRV detection	N.d.	-	N.d.	-
	40	Neg.	Neg.	N.d.	N.d.	N.d.	N.d.	-	N.d.	-
14	42	Neg.	Neg.	Neg.	N.d.	No HRV detection	N.d.	-	Neg.	Hospital admission

*Cov., reference genome coverage; Ct, cycle threshold values for real-time PCR (available for FTD and bacterial qPCR); HRV, human rhinovirus; mNGS, metagenomics by next-generation sequencing; Neg., negative; N.a., respiratory sample was not available; N.d., not done; *S. aureus*, *Staphylococcus aureus;* VP1, viral capsid-encoding gene.
†Patient 6 had an history of oxygen requirement, and metastatic bladder cancer.
‡Patient 9 was anorexic and on an intravenous drip.
§Patient 11 recently developed anorexia and cough.

Appendix Table 3. Set of primers used in the present study for human rhinovirus genotyping and human rhinovirus B14 RNA detection by real-time PCR

Sequencing / Real-time PCR	Primer/probe name	Sequence (5′-3′)	Size (bp)	Source
Sequencing	HRV_B_external_F	GATGAATTGGAAGAAGTCATTRTYGANAAA	30	(*14*)
Sequencing	HRV_B_external_R	CATCATCATGTGAGTAACCATCATARAA	28	(*14*)
Sequencing	HRV_B_internal_F	GAGAGCTTCTTGGGTAGAKCNGCNTGTGT	29	(*14*)
Sequencing	HRV_B_internal_R	CGTGCACCTGGAGGTACATACATRGCYTG	29	(*14*)
Sequencing	Fwd1a 941–961	AGTACCTTCCAGATGTAGAC	20	In-house designed
Sequencing	Fwd1b 961–981	GCCAGTGATGTCAACAAAAC	20	In-house designed
Sequencing	Rev1a 1874–1895	GAGTGGTTTTAAAGACCCCAT	21	In-house designed
Sequencing	Rev1b 1914–1934	CAGACCAGTGTGTGTAGTAC	20	In-house designed
Sequencing	Fwd2 1861–1882	TTGTTTATTGGTGATGGGGTC	21	In-house designed
Sequencing	Rev2a 3494–3512	CACAGTCTCCTGGCTCTG	18	In-house designed
Sequencing	Rev2b 3554–3572	ATCCACTGCCTCCAGCTG	18	In-house designed
Sequencing	Fwd3 3581–3602	TTGCTGATATACGACAATTGG	21	In-house designed
Sequencing	Rev3 3854–3876	ATGTACATTTTCAAAAACCTCC	22	In-house designed
Sequencing	Fwd4a 4021–4039	GTACTCCCCCAGGCCAAG	18	In-house designed
Sequencing	Fwd4b 4041–4063	AAAGCTGGAATTTTGCAGTAAA	22	In-house designed
Sequencing	Rev4a 4520	ATTATCTAGACTAGCCATTGGT	22	In-house designed
Sequencing	Rev4b 4563	AGGACTTAGGGTGTTGGAATT	21	In-house designed
Sequencing	Fwd5 4720	TGTGCAAAGATTGCCATCAAC	21	In-house designed
Sequencing	Rev5 6487	AGACAAACAAACCAAACTGGT	21	In-house designed
Real-time PCR	HRV_B14_F1	GACAAACTTGTTTATTGGTGAT	22	In-house designed
Real-time PCR	HRV_B14_R1	RAATCTAAGTGATCSAGACCA	21	In-house designed
Real-time PCR	HRV_B14_Probe1	AGTACTGAACAATTTCACCYAGAA	24	In-house designed
